# The role of artificial intelligence and deep learning in determining the histopathological grade of pancreatic neuroendocrine tumors by using EUS images

**DOI:** 10.1097/eus.0000000000000113

**Published:** 2025-05-02

**Authors:** Sercan Kiremitci, Gulseren Seven, Gokhan Silahtaroglu, Koray Kochan, Serife Degirmencioglu Tosun, Hakan Senturk

**Affiliations:** 1 Department of Gastroenterology, Bezmialem University School of Medicine, Istanbul, Turkey; 2 Management Information Systems Department, School of Business and Management Science, Istanbul Medipol University, Istanbul, Turkey.

**Keywords:** Artifical intelligence, Deep learning, EUS, Grade, Pancreatic neuroendocrine tumors

## Abstract

**Background and Objectives:**

Pancreatic neuroendocrine tumors (pNETs) are relatively rare and consist of 2% of the all pancreatic tumors. Although some of pNETs have a benign, nonprogressive course, some may be progressive and result with metastasis. We aimed to estimate the grade of pNETs by using artificial intelligence (AI) via deep learning (DL) algorithms as indexing to cyto/histopathological classification according to the World Health Organization 2017.

**Methods:**

A total of 803 EUS images were collected from 44 patients who had a cyto/histo-pathologically confirmed diagnosis with EUS fine-needle aspiration or biopsy (FNA/B). First, raw EUS images were prepared for processing by AI via DL algorithms, and convolutional neural networks were utilized to train the machine to predict the grades from EUS images. IBM SPSS 25.0 program was used for statistical analyses.

**Results:**

Thirty of the 44 patients (68%) were female, with a median age of 61 (range, 16–80) years. pNETs were mostly located in the pancreatic head: 24 cases (55%). Location was the neck in 3 (7%), body in 10 (22%), and tail in 7 (16%) patients. According to EUS-FNA/B results, 27 patients were grade 1 (G1) (61%); 12, grade 2 (G2) (27%); and 5, grade 3 (G3) (12%). In reference to the performance of AI for predicting the pathological grade, sensitivity was 94.29%; specificity, 97.14%; and accuracy, 96.19%. When the patient groups were subanalyzed as G1, G2, and G3 by the AI model to predict the pathological grade, the accuracy was as follows: for G1, 93.15%; for G2, 91.61%; and for G3, 98.05%.

**Conclusions:**

This pilot study suggests that pNET grade prediction can be reliably done on EUS images using AI-based technology.

## INTRODUCTION

Neuroendocrine neoplasms (NENs) originate from neuroendocrine cells and mainly localized in the lungs, gastrointestinal tract, and pancreas.^[1]^ Pancreatic neuroendocrine tumors (pNETs) are a relatively rare group of pancreatic tumors, accounting for approximately 2% of all pancreatic neoplasms, with an incidence of approximately 1/100,000.^[2]^ pNETs are classified into 2 groups―functioning and nonfunctioning―according to specific hormone secretion, with 60%–90% being nonfunctioning.^[3]^

In 2006, the European Neuroendocrine Tumor Society (ENETS) introduced the first classification for grading pNETs, which was based on the mitotic rate per 10 high-power fields (HPFs) and Ki-67 proliferative index.^[4]^ Subsequently, the 2010 World Health Organization (WHO) classification of pNETs was based on the ENETS classification: grade 1/grade 2 (G1/G2) pNETs were classified as well-differentiated neoplasms; and grade 3 (G3) pNETs were classified as poorly differentiated.^[5]^

In 2017, based on specific histopathological criteria to better predict biological behavior of the tumor, the WHO divided pancreatic NENs into 2 main categories: well-differentiated tumors, defined as G1 pNETs (<2 mitoses per 10 HPFs and/or a Ki-67 proliferation index <3%), G2 pNETs (between 2 and 20 mitoses per 10 HPFs or a Ki-67 proliferation index between 3% and 20%), and G3 pNETs (>20 mitoses per 10 HPFs or a Ki-67 proliferation index >20% without poorly differentiated features) and poorly differentiated pancreatic neuroendocrine carcinoma (pNEC), referring to G3 pNEC with >20 mitoses per 10 HPFs or a Ki-67 proliferation index >20% with poorly differentiated small or large cell features.^[6]^

Currently, surgery is the recommended approach for the treatment of pNETs. However, most small, low-grade tumors are nonprogressive, and expectant management may be an option. Therefore, accurate tumor grading is a pivotal component of management strategies.

EUS is an effective modality for identification of lesion characteristics, and sampling (fine-needle aspiration [FNA] or biopsy [FNB]). However, FNA/B may not yield sufficient material in every case. In a study, the diagnostic adequacy of EUS-FNA was tested in resected pNETs, measuring <2 cm in size, indexed to the pathology of resection. An accurate mitotic index in FNA samples was found in only 26.4% and the Ki-67 proliferation index in 20.1%.^[7]^ In another study evaluating EUS-FNA adequacy, the overall sampling accuracy was calculated as 74.7%, and it was emphasized that the EUS-FNA sampling adequacy was lower in pancreatic masses <2 cm (*P* = 0.04).^[8]^ In this context, various studies have been published on alternative methods for grade determination of pNETs.

Studies aimed at predicting the behavioral patterns and histopathological features of tumors based on the radiological features of pNETs were published.^[9,10]^ The preoperative grading of pNETs^[11]^ and prediction of Gleason score in prostate cancer^[12]^ using machine leaning (ML) models based on cross-sectional images were remarkable. Hitherto, no study was published, investigating the prediction of grading in pNETs using deep learning (DL) algorithms based on EUS images.

The present study aims to investigate whether artificial intelligence (AI) via DL algorithms predict pNETs grade according to the 2017 WHO classification by generating training and test groups over EUS images.

## METHODS

Data from patients, who underwent EUS for pancreatic lesions between 2012 and 2022 and diagnosed with pNETs using EUS-FNA/B, from our institution were retrospectively analyzed. Forty-seven patients were cyto/histopathologically diagnosed with pNETs during this period, and suitable-for-use EUS images were obtained from 44. A 22-gauge (Ga) FNA needle was used in 32 patients, 25-Ga FNA in 3, 19-Ga FNA in 3, and 22-Ga FNB in 6 patients (EUS-FNA ECO needle and EUS-FNB Trident™ needle; Micro-Tech Medical, Nanjing, China). Each pathology report was reviewed in detail by a pathologist and was graded as G1, G2, or G3 according to the 2017 WHO grading classification for pNETs.

In our endoscopy unit, written informed consent form is obtained from all patients to use the visual data anonymously for scientific studies, and this study was approved by ethics committee of our university (approval number: E-54022451-050.04-139636, date: February 2, 2024).

### Analysis of EUS features

All EUS examinations were performed using a linear echoendoscope (EG-3870UTK and EG38-J10UT Linear-Array Ultrasound Gastroscope; Pentax Europe GmbH, Hamburg, Germany). Digital reports of the patients were obtained from the endoscopy database and were reviewed by a single experienced endosonographer (HS) who knew that the lesions were pNETs but blinded to the EUS-FNA/B and surgical resection pathology results.

**Figure 1:**
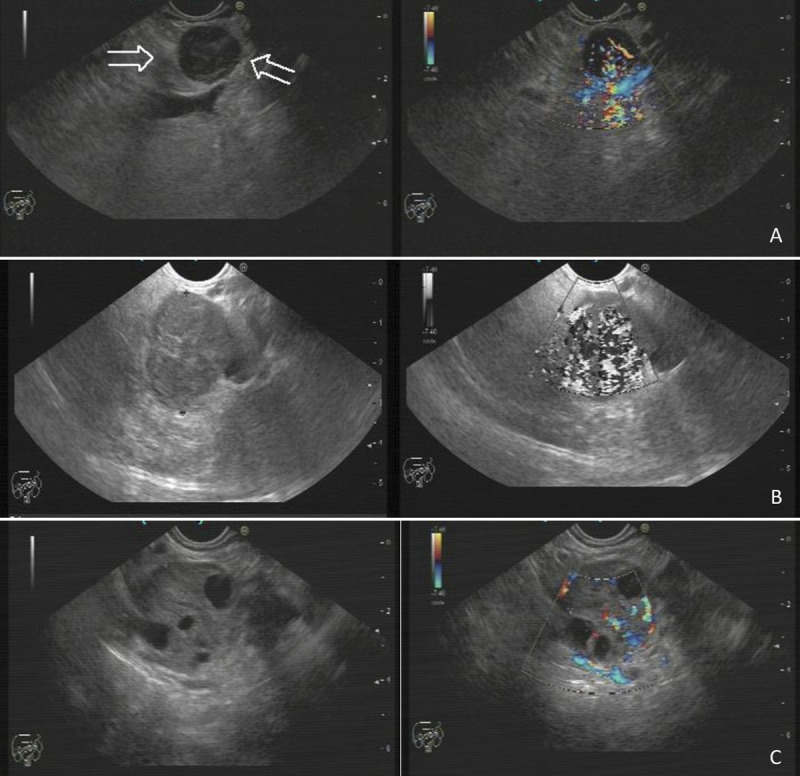
EUS images of G1 (A), G2 (B) and G3 (C) pNETs. A, G1 pNET: a round hypoechoic lesion in the body of pancreas measuring 13 mm. Borders are definite. It is hypoechoic and homogeneous with vascularity. Typical appearance of a small pNET. B, G2 pNET: an isoechoic lesion measuring 22 mm in the tail of pancreas. It is lobulated and borders are not well defined. It is relatively homogeneous and vascular. C, G3 pNET: an isoechoic, solid/cystic mass measuring 25 mm in the head of pancreas. Borders are quite definitive. It is heterogenous with cystic degeneration, and it is vascular. pNETs, pancreatic neuroendocrine tumors.

The following features were recorded for each tumor: size (mm), location (head, neck, body or tail), contour (round, oval, lobulated or irregular), border (well-defined or ill-defined), homogeneity (homogeneous or heterogeneous), echogenicity (hypoechoic, isoechoic or hyperechoic), cystic/solid state (cystic, solid or mix), vascularization (hypervascular or non-hypervascular), and presence or absence of pancreatic duct dilatation, vascular invasion, lymphadenopathy, calcification, and extraparenchymal lesion. Examples of EUS images of G1, G2, and G3 pNETs are shown in Figure 1.

### Artificial intelligence model

#### Data collection

Eight hundred three EUS images had been collected from our institution between 2012 and 2022. The dataset comprises images from 44 patients, of whom 27 were diagnosed as G1, 12 as G2, and 5 as G3 with EUS-FNA/B.

Surgical resection was performed on 36 (82%) out of 44 patients diagnosed with pNETs by EUS-FNA/B, and there was a significant correlation between EUS-FNA/B and resection pathology in reference to Ki-67 indexes. Therefore, we took EUS-FNA/B staging as a reference, and we served the EUS images of pNETs to the ML model for grade prediction.

#### Data preparation

Before forming the AI model and feeding the pipeline with images, some data preparation steps have been carried out. These steps were explained briefly below:

a) Initially, all images underwent normalization by dividing their values by 255. Normalization aims to rescale pixel values to a range of either 0 to 1 or −1 to 1. In this case, dividing by 255 achieves a range of 0 to 1. Alternatively, a range of −1 to 1 can be obtained by subtracting 0.5 and multiplying by 2. The main purpose of normalization is to reduce the impact of variations in image appearance, such as differences in lighting conditions, contrast, and color saturation, which may lead to the model learning irrelevant or misleading features. By normalizing pixel values, the model can focus more on significant image features, resulting in a more effective training process.
b) After that, Huang's Global Threshold method was applied to distinguish between foreground and background of an image. Either the threshold had been computed or manually chosen. The algorithm marks any pixel with an intensity value greater than this threshold with “1” and any pixel with intensity lower than the threshold as “0.” The result is a binary image that subsequently can be processed with the Connected Component Analysis Node to extract a labeling with separated segments. The Huang algorithm, also known as the maximum entropy thresholding algorithm, is a method for computing a global threshold for image segmentation. The algorithm is based on the principle of maximum entropy, which states that the best threshold is the one that maximizes the entropy of the “thresholded” image.^[13]^
c) Thirdly, using Lanczos interpolation, images were downsized to 28 × 28 × 1 dimension. Lanczos interpolation is a type of image interpolation method that is commonly used in digital image processing. It is a high-quality interpolation method that can be used to scale images while preserving their sharpness and detail.^[14]^
d) Because there was no balance in the number of G1, G2 and G3 diagnosed data, augmentation has been applied. This is done to increase the number of minority classes. For this purpose, the synthetic minority over sampling technique (SMOTE) has been used through 5 nearest neighbor hyperparameters. The basic idea behind SMOTE is to create new synthetic samples for the minority class by interpolating between existing samples. Specifically, SMOTE works by selecting a sample from the minority class and then choosing one of its k nearest neighbors randomly.^[15]^ Following this procedure, the total number of samples was increased to 1515.
e) During the division of image data into training and testing groups, a 10-fold cross-validation technique was employed, along with stratified sampling. The data were divided such that 70% was used for training the convolutional neural network (CNN) model, and the remaining 30% was used for testing the trained model and evaluating its performance.

**Figure 2:**
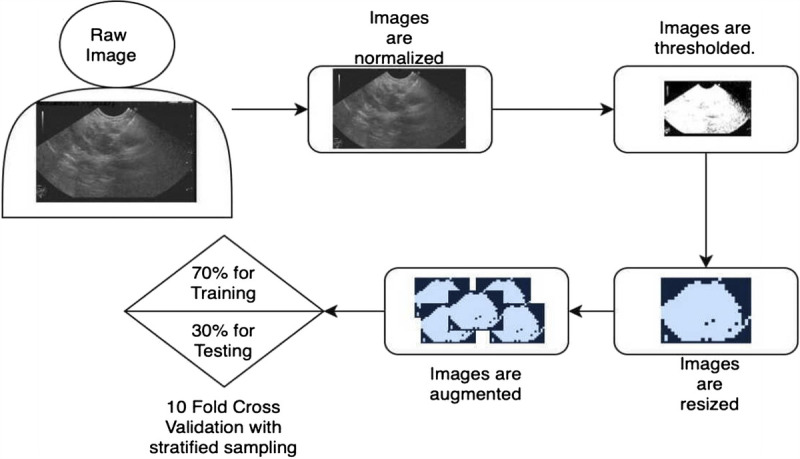
Data preparation process for artificial intelligence model.

Data preparation process for AI model is shown as a flowchart in Figure 2.

Although there were only 44 patients, at the beginning we had 803 images for training. Each image has been taken a unique case by the CNN model. However, because there was an imbalance in terms of grades, the SMOTE was used. After this process, the number of samples increased to 1515. Furthermore, we created a balance in terms of grades, with 505 images assigned to each label: G1, G2, and G3. We also used dropout as a hyperparameter to prevent possible overfitting.

In our study, only pNETs were graded, filed separately and subjected to ML model. The tumor's EUS images were provided raw to the AI model, and after completing the processes detailed in Figure 2, the images were prepared for use. The distinction between normal pancreatic parenchyma and tumors was not made separately, as tumors' EUS images were filtered and presented clearly to the ML model.

### Model used

The CNN, an improved version of artificial neural networks (ANN), was used as a DL algorithm in the study.^[16]^ CNN has been used to train the machine to distinguish and predict G1, G2, and G3 from EUS images. The CNNs are mostly used for image processing and computer vision. Convolutional networks use a mathematical operation that generates a third function using the other 2 functions. The new function defines how the shape of one function is modified by the other. The CNN contains multiple layers and hyperparameters that are necessary for training.^[17]^ Firstly, input images (pixel values) are sent to the convolution layer as (number) × (width) × (height) × (depth). Here a user-defined number of kernels produce feature maps. The kernel is another matrix that has a K value as (image width) × (image height) × (image depth).^[18]^ After kernel operations are completed, the produced matrixes are sent to pooling layers in order to rationalize or update computations. After updating, all productions may be used to feed another convolutional layer, and all operations are repeated in the next layer(s). Finally, the completed model will generate a new array in order to feed a regular ANN model. This process is known as flattening.^[19]^

Flattened data are inputs for ANNs that have their own fully connected hidden layers and neurons. Optimizer, learning rate, loss function, weight initialization, dropout rate are very important hyperparameters to be considered. An *optimizer* is an algorithm used to adjust the parameters, that is, weights of a CNN model during training to minimize the *loss function*, which is a mathematical function that measures the error between the model's predicted output and the actual target values. The optimizer determines how the weights are updated based on the gradients of the loss function with respect to the parameters.^[18]^ In this paper, *the adaptive moment estimation (Adam)* optimizer algorithm has been used. *Adam* is reported to have advantages because it utilizes adaptive learning rates for each parameter.

The *learning rate* is a hyperparameter that determines the size of the steps the optimizer takes when updating the model's weights. Smaller steps result in slower learning, but the results tend to be more reliable, as there is a lower chance of getting stuck in local minima during training.

The *loss function* used in this study is the well-known *mean squared error* (*MSE*), which measures the squared difference between predicted and actual values.^[18,19]^

Before learning begins, the model requires *initial weights*. Traditionally, weights are initialized randomly or arbitrarily. However, research suggests that proper weight initialization can lead to more effective learning and faster convergence. In this paper, *Xavier/Glorot Initialization* has been employed instead of random initialization. Xavier/Glorot Initialization aims to maintain consistent variance of activations and gradients across layers.^[20]^

Overfitting is a critical concern in ML. To mitigate this, *dropout* is used in CNNs. The *dropout rate* refers to the proportion of neurons dropped during training. For instance, a dropout rate of 0.1 means that 10% of the neurons in the layer are randomly ignored during each forward and backward pass. This drives the model to exercise on different subsets of features; this helps in enhancing its generalization ability.^[19]^

Although they play very important roles in the success of training, there is not a rule of thumb to determine them beforehand. They are often adjusted by trial-and-error.^[21]^

**Figure 3:**
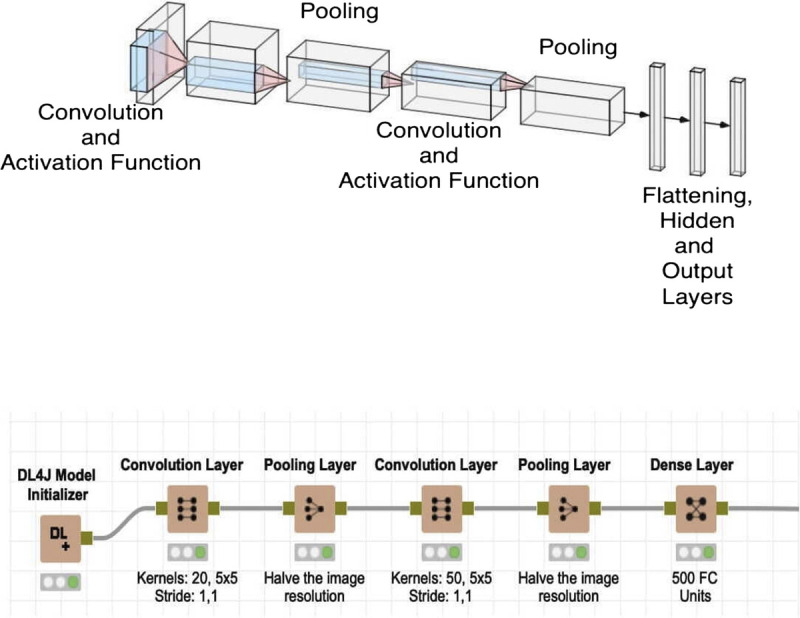
Flattening process and convolutional neuronal network model.

In this study, flattening process and CNN model have been formed as it is depicted in Figure 3.

CNNs identify subtle patterns, textures, and spatial relationships that may not be apparent to human observers. However, they do not explicitly analyze surrounding tissue changes or vascular features. Although the exact mechanisms of their decision-making are not fully understood, it is likely that texture, shape, margins, and intensity are among the key factors considered by CNNs.

### Survival analysis

Survival analysis was performed to determine the potential of the pNETs pathological grade classification in survival prediction. Patients were divided into the G1 and G2/G3 groups. The Kaplan-Meier (KM) method was used for the survival analysis of G1 and G2/G3 pNETs. Overall survival (OS) was defined as the time from the date of diagnosis to the time until death.

### Statistical analysis

IBM SPSS 25.0 program was used for statistical analyses. Descriptive statistical methods (median, frequency, percentage) were used to evaluate the study data. The conformity of quantitative data to normal distribution was evaluated by the Kolmogorov-Smirnov test and Shapiro-Wilk test. Mann-Whitney *U* test was used to compare quantitative variables that did not show normal distribution between the groups. The chi-square test and Fisher's exact test were used to compare qualitative data. Patients were divided into 2 groups, G1 and G2/G3, based on EUS-FNA/B results, and the effect of tumor grade on survival in pNETs patients was analyzed using KM survival analysis. A value of *P* < 0.05 was considered as significant.

## RESULTS

### Patient characteristics

Between 2012 and 2022, 47 patients who were evaluated using EUS with a referral diagnosis of pancreatic lesions were diagnosed with pNETs, in whom 44 suitable EUS images were available for use in both the training and test groups. Thirty of the 44 (68%) patients were female, and the median age was 61 years (range, 16–80 years). The pNETs were classified as functional in 10 (22%) patients, and all were insulinomas: 4 were located in the pancreatic head, 3 in the body, and 3 in the tail. During follow-up, 36 (82%) patients underwent resection, with 33 (91%) confirmed to have pNETs according to surgical pathology examination. A pathological study of the resected lesions revealed non-pNETs in 3 patients; serous cystadenoma in 2, and paraganglioma in 1. Based on the resection results, 11 patients were diagnosed with G1 pNETs and 22 with G2 pNETs. During a median of 176 weeks (range, 44–401) of follow-up, 7 out of 44 (16%) patients expired due to pNETs related complications. Baseline patient characteristics are summarized in Table 1.

**Table 1:** Patient characteristics.

		(*n* = 44)		
Age (yr)		61 (16–80)*		
Follow-up period (wk)		176 (44–401)*		
Gender	Female	30	68%	
	Male	14	32%	
Localization	Head	24	55%	
	Neck	3	7%	
	Body	10	22%	
	Tail	7	16%	
Insulinoma		10	22%	
EUS size (mm)		20 (7–80)*		
EUS Ki-67 (%)		3 (1–80)*		
EUS, grade	G1	27	61%	
	G2	12	27%	
	G3	5	12%	
Metastasis		11	25%	
Metastasis location	Liver	4	10%	
	Lymph node	6	13%	
	Liver + lymph node	1	2%	
Status	Under follow-up	37	84%	
	Expired	7	16%	

*Median (range).

G, grade.

### Association of EUS features between selected groups

Lesions with an EUS size of ≤2 cm had a lower risk of metastasis (*P* = 0.001), more likely to be located in the body-tail (*P* = 0.039), and more homogeneous (*P* = 0.003) than lesions >2 cm. Statistical analyses are shown in Table 2.

**Table 2:** Comparison of EUS and clinical features between ≤2- and >2-cm pNETs.

		EUS size ≤2 cm (*n* = 29, 65%)		EUS size >2 cm (*n* = 15, 35%)		*P*
Metastasis		2	7%	9	60%	0.001*
Localization	Head-neck	15	51%	12	80%	0.835^†^
	Body-tail	14	49%	3	20%	0.039*
Homogeneity	Homogenous	24	83%	7	46%	0.003^†^
	Heterogenous	5	17%	8	54%	

*Fisher's exact test.

^†^Chi-square test.

pNETs, pancreatic neuroendocrine tumors.

Image characteristics of G1 and G2/G3 pNETs were compared, but no significant difference was found and results are revealed in Table 3.

**Table 3:** Comparison of EUS features between G1 and G2/G3 pNETs.

		EUS Grade 1 (*n* = 27, 61%)		EUS Grade 2/3 (*n* = 17, 39%)		*P*
EUS size (mm)		17 (7–80)*		21,50 (10–60)*		0.081^†^
Localization	Head	17	63%	7	41%	0.122^‡^
	Neck	1	4%	2	12%	0.549^§^
	Body	5	18%	5	29%	0.726^§^
	Tail	4	15%	3	18%	0.696^§^
Shape	Round	21	76%	9	53%	0.147^‡^
	Oval	2	8%	4	24%	0.186^§^
	Lobulated	2	8%	1	6%	1.000^§^
	Irregular	2	8%	3	17%	0.662^§^
Border	Well-defined	18	67%	11	65%	0.750^‡^
	Ill-defined	9	33%	6	35%	
Homogeneity	Homogeneous	20	74%	11	65%	0.869^‡^
	Heterogeneous	7	26%	6	35%	
Echogenicity	Hypoechoic and isoechoic	24	88%	14	82%	0.255^§^
	Hyperechoic	3	12%	3	18%	
Cystic/Solid component	Solid	24	88%	12	70%	1.000^§^
	Cystic and mix	3	12%	5	30%	
Vascularization	Hyper vascular	10	36%	8	46%	1.000^§^
	Non-hyper vascular	5	18%	4	23%	
Pancreatic duct dilatation	Yes	0	0%	1	6%	0.383^§^
	No	27	100%	16	94%	
Vascular invasion	Yes	1	4%	1	6%	1.000^§^
	No	26	96%	16	94%	
Lymphadenopathy	Yes	0	0%	1	6%	0.383^§^
	No	27	100%	16	94%	
Tumoral calcification	Yes	3	11%	1	6%	1.000^§^
	No	24	89%	16	94%	
Extra parenchymal lesion	Yes	0	0%	1	6%	0.383^§^
	No	27	100%	16	94%	

There are no data regarding vascularity in 17 out of 44 patients on EUS feature evaluation.

*Median (Range).

^†^Mann-Whitney *U* test.

^‡^Chi-square test.

^§^Fisher's exact test.

G, grade; pNETs, pancreatic neuroendocrine tumors.

### Correlation analysis between EUS-FNA/B and surgical pathology

Thirty-six (82%) patients underwent resection, and the surgical pathology result was reported as pNETs in 33 (91%) of them. There was a significant correlation between EUS-FNA/B and surgical pathology Ki-67 indexes (spearman correlation coefficient: 0.502, *P* = 0.003), as well as measured by EUS and in surgically resected tumor sizes (Spearman correlation coefficient: 0.852, *P* < 0.001).

### Prediction of pathological grading of pNETs using AI model

The overall sensitivity, specificity, positive predictive value (PPV), negative predictive value (NPV), and accuracy of AI for predicting and distinguishing the pathological grade of pNETs using EUS images were 94.29% (95% CI, 91.74%–96.23%), 97.14% (95% CI, 95.84%–98.13%), 94.29% (95% CI, 91.86%–96.02%), 97.14% (95% CI, 95.90%–98.02%), and 96.19% (95% CI, 95.03%–97.14%), respectively. As the cases were divided into G1, G2, and G3 groups, using the AI model to predict the pathological grade of pNETs, the sensitivity, specificity, recall, accuracy, and F-measure were 89.47%, 96.7%, 89.47%, 93.15%, and 91.28% for G1 pNETs, respectively. The sensitivity, specificity, recall, accuracy, and F-measure of AI for predicting the pathological grade of G2 pNETs were 93.42%, 95.71%, 93.42%, 91.61%, and 92.51%, respectively. The sensitivity, specificity, recall, accuracy, and F-measure of AI for predicting the pathological grade of G3 pNETs were 100%, 99.01%, 100%, 98.05%, and 99.02%, respectively. The EUS-FNA/B grade was used as the reference for this evaluation, and the data are summarized in Table 4.

**Table 4:** Performance of artificial intelligence in grading.

	Sensitivity	Specificity	NPV	PPV	Accuracy
Overall	94.29%	97.14%	97.14%	94.29%	96.19%
G1 pNETs	89.47%	96.7%	96.1%	89.47%	93.15%
G2 pNETs	93.42%	95.71%	95.1%	93.42%	91.61%
G3 pNETs	100%	99.01%	99.02%	100%	98.05%

G, grade; NPV, negative predictive value; pNETs pancreatic neuroendocrine tumors; PPV, positive predictive value.

### Survival analysis

**Figure 4:**
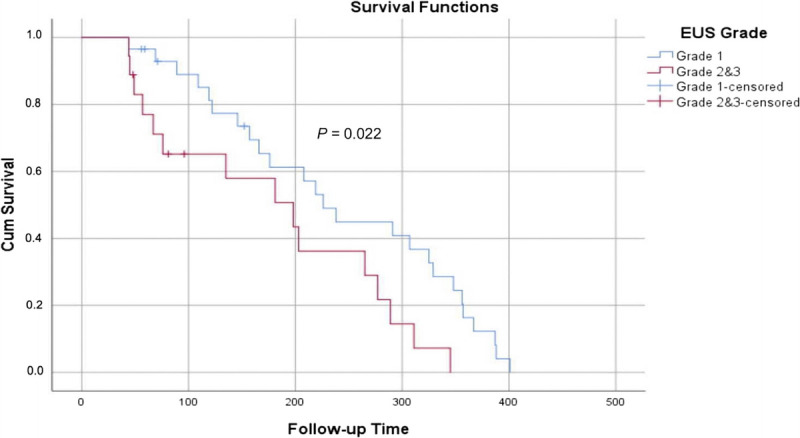
Kaplan-Meier survival analysis and long-rank test for G1 *versus* G2/G3 pNETs.

Survival of patients was evaluated with KM survival analysis, and as expected, we found that the survival of G1 pNETs patients was significantly higher than the survival of G2/G3 pNETs (log-rank test, *P* = 0.022). The KM analysis is shown in Figure 4.

## DISCUSSION

To the best of our knowledge, this is the first study to predict the grade of pNETs using EUS images assessed by AI via DL algorithms. According to our results, AI is an effective tool for grading pNETs based on 2017 WHO classification. It determined the grade of pNETs with 94.29% sensitivity, 97.14% specificity, and 96.19% accuracy. In subanalyses according to the 2017 WHO classification, grade determination accuracy values were 93.15%, 91.61%, and 98.05% for G1, G2, and G3 pNETs, respectively.

EUS-FNA/B is one of the most commonly used methods for the histopathological diagnosis of gastrointestinal and pancreatic lesions.^[22]^ However, endoscopist, patient, and/or lesion-related factors may affect tissue adequacy. In 2016, a study by Hijioka et al. reported that 49 of 60 pNETs were correctly diagnosed using EUS-FNA, and in the multivariate analysis, tissue yield was significantly lower in tumors localized to the pancreatic head (*P* = 0.04) and tumor rich in stromal fibrosis (*P* = 0.03). However, tumor size, needle type, and presence of cystic components did not affect the yield.^[23]^ In 2022, Togliani et al. reported that tissue adequacy obtained by EUS-FNA/B was statistically less yielding in solitary lesions of the pancreas in the head–uncinate region with a higher fibrosis score.^[24]^

Another contentious issue is the consistency between EUS-FNA/B and surgical pathology results. We found a significant correlation between EUS-FNA/B and resection pathology Ki-67 indexes; however, this consistency was not observed in every series. In a study comparing the pathological results of patients who underwent EUS-FNB and surgery for pNETs, significant correlation was not found between EUS-FNB and resection Ki-67 indexes. It was found that the resection Ki-67 index was higher than that of EUS-FNB, and only 2 of the 12 patients diagnosed with G2-pNETs according to EUS-FNB were confirmed in the resected tissue.^[25]^

EUS-FNA/B of in pNETs is mostly safe but rarely complicated with infection that may develop after the puncture of cystic lesions, bleeding from pseudoaneurysms, acute pancreatitis (AP), and tumor seeding. AP is mild-to-moderate in these cases but may require hospitalization. In our series, AP developed after EUS-FNA in 2 patients and 1 required hospitalization for 48 hours and the other for 72 hours. The rate of AP after EUS guided biopsy has been reported up to 9.2%, especially in patients in whom needle traverses >5 mm of the normal pancreatic parenchyma or penetrates pancreatic duct.^[26]^

Our results comparing ≤2- and >2-cm pNETs were similar to a multicenter international study by Maggino et al., that evaluated 263 resected cystic pancreatic neuroendocrine tumors (cpNETs), 63.5% of which were >2 cm in size. cpNETs >2 cm exhibited more aggressive behavior pattern compared with those ≤2 cm.^[27]^

However, in contrast to our results, some studies have reported that pNETs ≤2 cm in size may also exhibit an aggressive behavior pattern and may metastasize at a high rate. Gratian et al. evaluated 1854 nonfunctioning pNETs ≤2 cm in size and found that 29% had lymph node, and 10% had distant metastasis.^[28]^ These very large case series emphasized that pNETs ≤2 cm in size may not be as benign as generally thought and may be associated with a risk for metastasis.

Some other studies also reported that surgery, despite its risks, yields better OS in comparison to expectant management for ≤2-cm pNETs. A study by Sharpe et al. included patients with pNETs ≤2 cm, 309 of which underwent resection, whereas 71 had been followed up. In patients who underwent resection, the median survival was >5 years, and the 5-year OS rate was 82.2%; in patients who were followed up, the median survival was 2 years, and the 5-year OS rate was 34.3%. In the KM analysis, the survival of patients who underwent resection for pNETS ≤2 cm was significantly better than those who were followed up (*P* < 0.0001).^[29]^

In our study, 29 of 44 patients had pNETs ≤2 cm in size, 5 of which did not undergo resection. In this context, although pNETs ≤2 cm exhibit benign features, and no patient who was followed up without resection died. These patients should be evaluated on a case-by-case basis, and decision regarding surgery should be based on individual risk factors as well as patients' opinion.

Various studies evaluated pancreatic lesions using AI via DL algorithms with magnetic resonance imaging (MRI) or CT images. Some used histopathological data, and some used microRNA or gene expression assay data for the prognostic and diagnostic course of pancreatic lesions.

According to a review by Pantelis et al., including 44 studies that evaluated gastroenteropancreatic neuroendocrine tumors by using AI and ML, 34% used imaging data; however, only 9% used EUS images as a source.^[30]^

Previous AI-based studies have generally focused on histological grade prediction, metastasis risk, and the differentiation of pancreatic lesions using CT and MRI imaging. A pioneering study used convolutional and long short-term memory neural network models to make differential diagnosis of 65 focal pancreatic lesions. In total, 2068 images were used for the training model, and 672 were used to test the DL models. The AI-based model was able to discriminate lesions with an overall accuracy of 98.2%. In subgroup analyses, pancreatic ductal adenocarcinoma, chronic pseudo-tumoral pancreatitis, and pNETs were diagnosed with accuracy values of 97.61%, 98.66%, and 98.51%, respectively.^[31]^

A study by Luo et al., which aimed to preoperatively predict and differentiate grades as G1/G2 or G3 for pNETs using AI-based DL modeling over CT image features, found that AUC of the arterial model was significantly higher than those of the venous and arterial/venous model (AUC = 0.81).^[32]^ Another study was designed to predict the grade of pNETs using AI-based MRI modeling. The MRI images were replicated using generative adversarial networks and CNN models, and imbalances between the images were eliminated. In reference to the performance of CNN, the mean accuracy was 85.13%, and the micro-average AUC was 0.9117 for the internal validation set; the mean accuracy was 81.05%, and the AUC was 0.8847 for the external validation set.^[33]^

These studies were highly robust in predicting the grade of pNETs using radiological images; however, none defined the accuracy for G1, G2, and G3 pNETs separately.

Unlike these previous studies, the first original outcome of our study was the ability of AI to predict grades for pNETs using EUS images. The second was to determine the sensitivity, specificity, PPV, NPV, and accuracy overall as well as G1, G2, and G3 grades separately with ML.

Our study has some limitations: It has a retrospective design, and the sample size is relatively small that, after eligible EUS images were reproduced using the SMOTE method, 70% of the data were used as the training set and the remaining 30% were used as the validation set. In addition, not all lesions were sampled using the same type of EUS needle: Some lesions were sampled with FNA and some others with FNB of different sizes. Finally, the quality of the EUS images obtained from the imaging database may not be the same as that of real-time EUS examinations, or the image quality may have been compromised during transfer from the database.

## CONCLUSIONS

This pilot study suggests that pNET grade prediction by EUS images is poor. However, EUS-FNA/B sampling was robust. Similar or better prediction may be achieved, noninvasively, by using AI-based technology.

## Source of Funding

No funding was received for this study.

## Ethical Approval

This study was approved by ethics committee of our university (approval number: E-54022451 050.04-139636, date: February 2, 2024).

## Informed Consent

In our endoscopy unit, written informed consent form is obtained from all patients to use the visual data anonymously for scientific studies.

## Conflict of Interest

The authors declare that they have no financial conflict of interest with regard to the content of this research article.

## Author Contributions

Hakan Senturk designed the study, performed EUS procedures as part of the study, assisted writing the manuscript, and reviewed the manuscript. Sercan Kiremitci designed the study, collected and analyzed data, wrote the manuscript and drafted the article. Gokhan Silahtaroglu performed the required statistical analyses and conducted artificial intelligence model. Serife Degirmencioglu Tosun assisted in study and obtained ethical approval for the study. Koray Kochan and Gulseren Seven had worked on data collection and extraction. Hakan Senturk, Sercan Kiremitci, Gulseren Seven, Gokhan Silahtaroglu, Koray Kochan, Serife Degirmencioglu Tosun did final approval of the version to be submitted. This article has been read and approved by all the authors and the requirements for authorship have been met.

## Data Availability Statement

Data described in the manuscript will be made available from the corresponding author on reasonable request.
